# Inflammation Predicts Decision-Making Characterized by Impulsivity, Present Focus, and an Inability to Delay Gratification

**DOI:** 10.1038/s41598-019-41437-1

**Published:** 2019-03-20

**Authors:** Jeffrey Gassen, Marjorie L. Prokosch, Micah J. Eimerbrink, Randi P. Proffitt Leyva, Jordon D. White, Julia L. Peterman, Adam Burgess, Dennis J. Cheek, Andreas Kreutzer, Sylis C. Nicolas, Gary W. Boehm, Sarah E. Hill

**Affiliations:** 1 Texas Christian University, Department of Psychology, 2955S University Dr, Fort Worth, TX United States of America; 2 Texas Christian University, Harris College of Nursing and Health Sciences, 2800 W Bowie St, Fort Worth, TX United States of America; 3 Texas Christian University, Department of Kinesiology, 2800 W Bowie St, Fort Worth, TX United States of America

## Abstract

Here, we propose a novel theoretical model linking present-focused decision-making to the activities of the immune system. We tested our model by examining the relationship between inflammatory activity – *in vivo* and *in vitro* – and decision-making characterized by impulsivity, present focus, and an inability to delay gratification. Results support our model, revealing that inflammation predicts these outcomes even after controlling for factors that may contribute to a spurious linkage between them. Moreover, subsequent analyses revealed that our model was a better fit for the data than alternative models using present-focused decision-making and its health-harming behavioural sequelae (e.g., smoking, risky sexual behaviour) to predict inflammation, lending support for the proposed directionality of this relationship. Together, these results suggest that inflammation may contribute to decision-making patterns that can result in undesirable personal and societal outcomes.

## Introduction

The ability to delay gratification plays a critical role in determining a number of important life outcomes, such as educational attainment^1^, accumulation of wealth^2^, social functioning^3^, and health^4^. Lacking this ability therefore represents a major risk factor for many undesirable outcomes. For example, research finds that impulsivity – a construct characterized by present focus and an inability to delay gratification – is a significant predictor of substance abuse, problem gambling, and risky sexual behaviours^5,6^. Such behaviours have serious social and economic consequences. In the United States alone, excessive alcohol consumption and tobacco use are estimated to bear annual health costs of over $249 billion and $170 billion, respectively^7,8^. In examining the factors that contribute to an inability to delay gratification, much of the research has focused on the role played by cues in the external environment, such as developmental stressors^9^, poverty^10^, and being in the presence of untrustworthy others^11^.

In the current research, we combine theoretical insights from the evolutionary sciences^12–14^ and psychoneuroimmunology^15–19^ to shift the focus inward, proposing that inflammation – which occurs in the context of illness or cellular distress – may play an important mechanistic role in the preference for immediate versus delayed rewards. Specifically, our model proposes that inflammation should increase the desire for immediately available resources, as this is a context in which both resource need^15,20^ and mortality risk^13,14^ are elevated. Although such a shift might favour healing and recovery in the short term, in the long term – when inflammation is protracted or chronic – the resulting behavioural sequelae may exacerbate inflammation, reinforcing further present-focused behaviours.

The trade-off between taking advantage of currently available opportunities and rewards or forgoing those opportunities to seek a better outcome down the road is a fundamental decision-making trade-off that occurs across species and decision domains^12^. Examining how individuals resolve this trade-off has thus been an important area of research for psychological disciplines as diverse as social psychology^21^, developmental psychology^22^, cognitive psychology^12^, learning^23^, and neuroscience^24^.

In the evolutionary sciences, the trade-off between choosing immediate versus delayed rewards is often examined using the framework of risk-sensitive foraging theory, or RSFT^25–28^. According to RSFT, whether an organism should take advantage of current resource opportunities or wait for better alternatives will depend on (a) the risk that delayed rewards may become unavailable (i.e., the collection risk) and (b) the opportunity costs associated with not having immediate resource access^28^ (see also life history theory^29–31^).

Decades of research has found support for these predictions. For example, individuals living in unpredictable environments (a context with elevated collection risk) often exhibit a preference for smaller, sooner rather than larger, later rewards^10,30,31^. Others find that contexts in which the opportunity costs of resource acquisition are particularly high (e.g., when the benefits of investing effort in mating or somatic repair are paramount) also increase the preference for immediately available resources^32,33^.

Among the variables that impact the risks and costs associated with waiting for later rewards is the internal, physical condition of the body^13,14,33^. If an individual’s body is in good condition (i.e., one is in good health), the individual is in less need of immediate resources to help manage a metabolically costly response to infection, injury, or disease^15,20^, and is more likely to survive into the future to realize delayed rewards (see also internal prediction models^13,14^). Alternately, if an individual is in poor health, more immediate rewards should be favoured over those available after a delay, as current energy demands are higher, and the future is relatively less certain.

Here, we build on these ideas, examining the role of signaling by the immune system in individuals’ preferences for immediate versus delayed rewards. When the cells of the immune system detect infectious agents or cellular distress, they release signaling proteins, such as proinflammatory cytokines, which coordinate biological events that help prevent or clear infections and heal injuries^34^. In addition to coordinating the activities of the immune cells themselves (e.g., white blood cells), cytokines also influence the activities of the nervous system^15–18,35^. For example, cytokines orchestrate sickness behaviour, the constellation of physical, psychological, and behavioural changes – such as anhedonia, diminished foraging, and social avoidance – that occur in the context of an acute immune response, in order to mitigate bodily damage from infection and conserve energy for use in immunological defence^15^.

Moreover, research suggests that inflammation also promotes psychological shifts that increase one’s sensitivity to cues of potential threats and opportunities in the environment when the body is in poor condition. For example, research in humans finds that experimental immune activation (i.e., using typhoid vaccination) leads to a rise in circulating interleurkin-6 (IL-6), which is accompanied by increased activity in brain regions associated with internal state monitoring (e.g., anterior insula)^36^. Other studies employing similar experimental designs have found that inflammation also has important implications for social perception. Specifically, this research finds that increases in inflammation lead to greater neural sensitivity to negative social experiences, such as social rejection^37^ and socially-threatening stimuli^38^, as well as to positive social experiences, such as pictures of support figures^39^.

**Figure 1:**
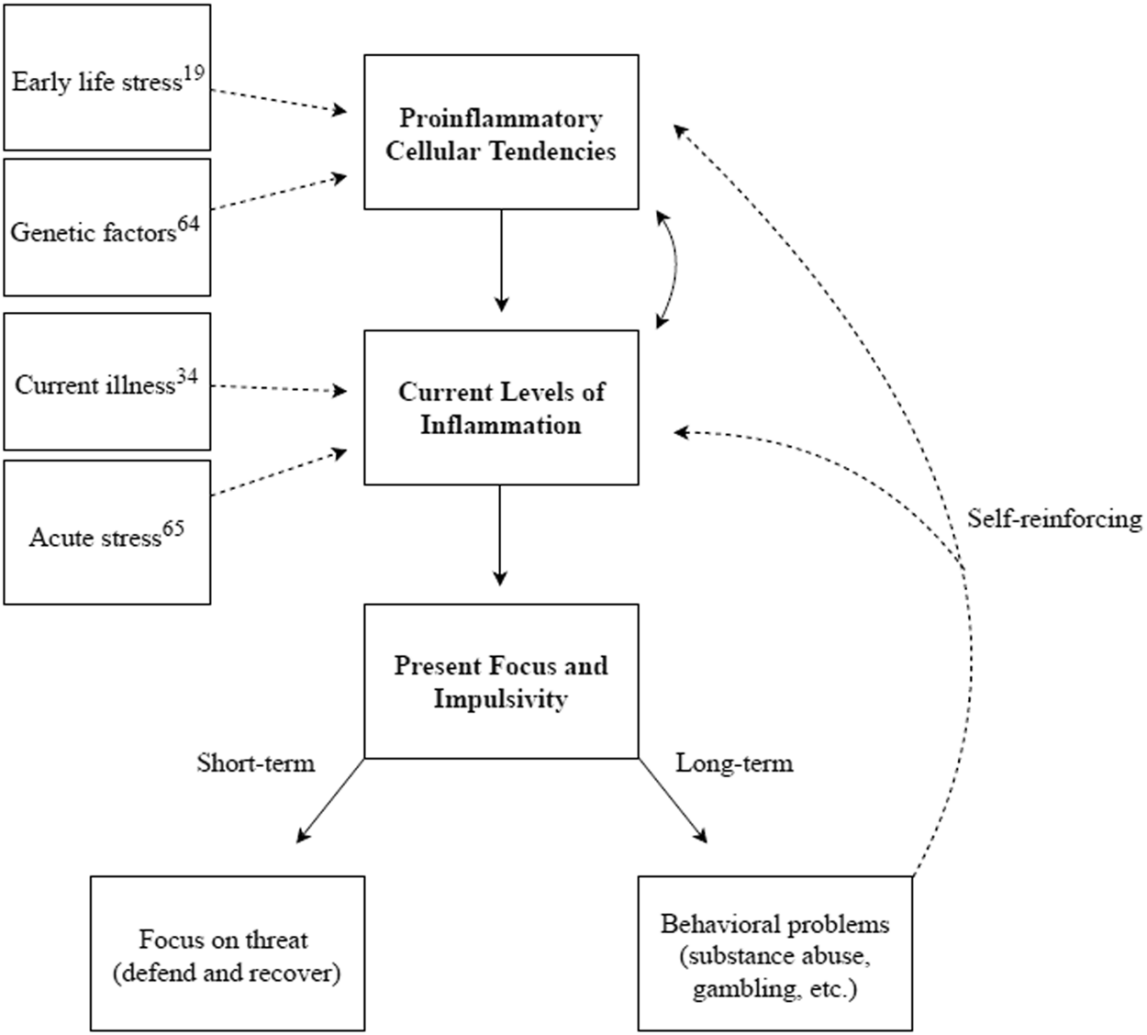
Model demonstrating the relationship between inflammation and present focus. Dashed lines represent factors known to predict inflammation (both the tendency toward inflammation, shown at top, and current inflammation, shown below) based on previous research. Unidirectional arrows suggest direct paths, while double-edged arrows suggest a bi-directional relationship.

Given that immunological activity (a) increases the body’s need for immediately available resources^15,20^, (b) indicates a relatively diminished probability of survival to reap later-available rewards^13–15^, (c) facilitates psychological and behavioural shifts that promote fitness accounting for *a* and *b*^15,36–39^, we hypothesized that inflammation would predict a more present – as opposed to future – focus and a greater desire for immediate gratification. We present our theoretical model in Fig. 1.

In the current research, we tested our model by examining the relationship between inflammation and present-focused decision-making in a sample of healthy human participants (*N* = 159; 80 men, 79 women; *M*_age_ = 20.17 years, *SD* = 2.75). The participants in our sample, in addition to being required to be free of illness for two weeks prior to testing, were instructed to abstain from behaviours such as smoking, drinking alcohol, and having sexual activity, for 48 hours prior to testing. This was done to minimize any effects of health-harming behavioural correlates of impulsivity on inflammation, which is a second pathway through which inflammation and impulsivity are related (see Fig. 1)^36–38^. Building on the results of pilot data (see Supplementary Information for details), recent research^40–42^ and emerging theoretical work^43^, we hypothesized that higher levels of inflammation – even when measured in healthy individuals and outside the context of acute illness – would predict present focus and a greater preference for immediate over delayed rewards. Consistent with the idea that inflammation plays a mechanistic role in promoting these decision patterns, we predicted that this relationship would be predicted directly by levels of inflammation present the day of the testing session (measured *in vivo*), but only indirectly by one’s cellular tendency to exhibit an exaggerated inflammatory response to an immune challenge presented *in vitro*. Finally, consistent with the proposal that inflammation predicts present focus (rather than the reverse), we predicted that alternative models testing for a reverse causal chain would not be supported.

If confirmed, the predicted results will lend key initial support for the hypothesis that inflammation – even at sub-clinical levels – plays an important role linking the internal, physiological condition of the body to present-focused decision-making and impulsive tendencies.

## Results

### Data analytic plan

**Figure 2:**
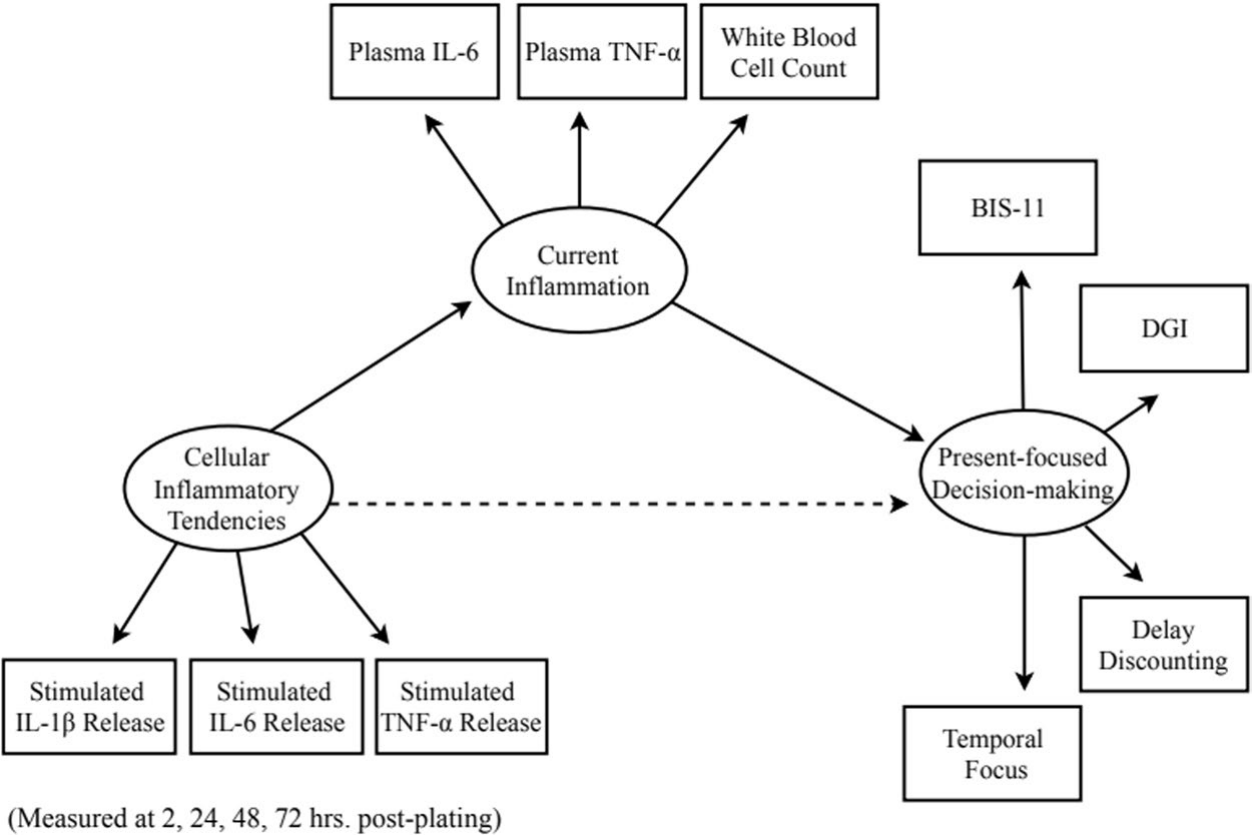
Hypothesized structural path model. We hypothesized that the latent construct of cellular inflammatory tendencies (*in vitro* release of IL-1β, IL-6, and TNF-α by PBMCs) would predict current inflammation (plasma levels of IL-6 and TNF-α, as well as white blood cell count), which would, in turn, predict present-focused decision-making (BIS-11, DGI, temporal focus, and delay discounting). We further hypothesized an indirect effect of cellular inflammatory tendencies on present-focused decision-making mediated through current inflammation. BIS-11 = Barratt Impulsiveness Scale; DGI = Delaying Gratification Inventory.

For our primary, hypothesized statistical model (see Fig. 2), lipopolysaccharide (LPS)-stimulated release of each cytokine by PBMCs *in vitro* was modelled as a latent factor of cellular inflammatory tendencies (i.e., IL-6, IL-1β, and TNF-α). Each *in vivo* inflammation measure (plasma levels of IL-6 and TNF-α, as well as white blood cell count) together comprised a latent factor of current inflammation. All measures of present-focused decision-making (delay discounting, temporal focus, self-reported impulsivity, and self-reported inability to delay gratification) together comprised a latent factor of present focus/impulsivity. We regressed the latent factor of current inflammation on the latent factor of cellular inflammatory tendencies, and regressed the latent factor of present focus/impulsivity on both current inflammation and cellular inflammatory tendencies. We conducted a post-hoc power analysis using Monte Carlo simulation to determine whether we achieved adequate power (i.e., 0.80 power) to reliably estimate each parameter in the model^44^. The results of this simulation can be found in the Supplementary Information.

Prior to testing the hypothesized model, we first examined the data to determine whether all assumptions for accurate estimation using structural equation modelling (SEM) were met (MPlus 7.4 statistical software)^45^. First, because plasma levels of both IL-6 and TNF-α – as well as white blood cell counts – were positively skewed, these values were log-transformed, per convention^45,46^, which corrected the distribution to approximate normality. All *in vitro* stimulated cytokine levels were also positively skewed. These values were square root-transformed, as log-transformations over-corrected the distributions to a negative skew.

Because the stimulated cytokine release scores contained a hierarchical structure with four time-points nested within each participant, we used the TYPE = COMPLEX command in MPlus, which allows for specifying each individual as a cluster. This statistical method allowed us to both account for dependence in the stimulated cytokine release data, as well as account for the slope of cytokine release over time in the analysis by including all values (i.e., at each time-point) in the cluster for each cytokine (i.e., IL-6, IL-1β, and TNF-α), rather than averaging across time-points.

Model fit was assessed using four fit indices: *χ*^2^ test of model fit, the comparative fit index (CFI), the root mean square error of approximation (RMSEA), and the standardized root mean square residual (SRMR). Adequate model fit was indicated by a non-significant *χ*^2^ value (*p* > 0.05), a CFI value >0.95, an RMSEA value <0.05, with the upper bound of the confidence interval less than 0.10, and an SRMR statistic <0.05. All significance tests were two-tailed. See Fig. 2 for our hypothesized statistical model and Table S2 in Supplementary Information for model fit statistics.

### Results of structural path models

**Figure 3:**
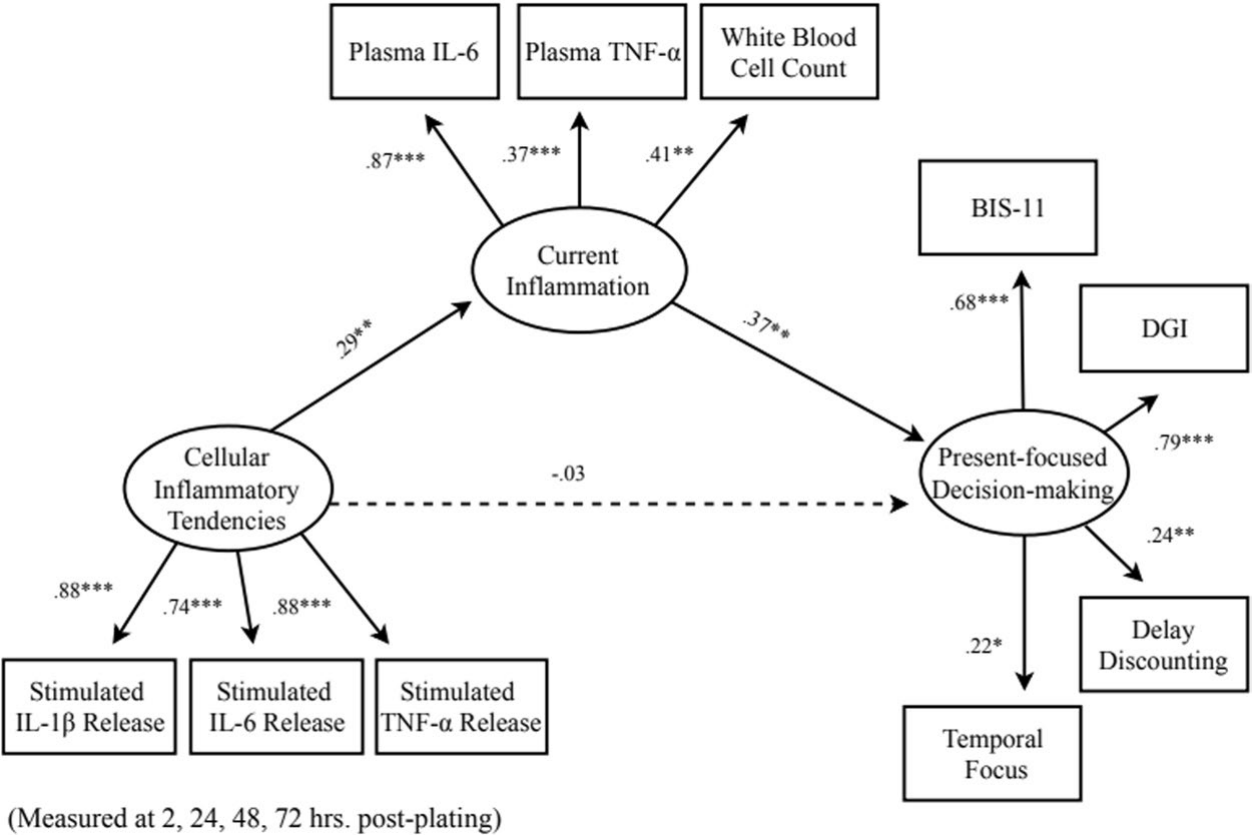
Final model shown with standardized estimates. Shown here are the results of the primary model without controlling for covariates. Results of the model controlling for covariates can be found in the Supplementary Information. It should be noted that the model controlling for covariates was a poor fit to the data. Dotted lines denote non-significant paths. BIS-11 = Barratt Impulsiveness Scale; DGI = Delaying Gratification Inventory. ***p < 0.001; **p < 0.01; *p < 0.05.

See Table 1 for Descriptive Statistics. First, we tested our hypothesized path model. Fit statistics for this model revealed good fit (see Table S2 in Supplementary Information). Results revealed that individuals whose PBMCs exhibited more *in vitro* cytokine release in response to simulation with LPS (i.e., individuals showing greater proinflammatory tendencies) also exhibited higher plasma levels of inflammation (*β* = 0.29, *SE* = 0.10, *t* = 2.79, *p* = 0.005). In turn, higher plasma levels of inflammation predicted greater present-focused decision-making (*β* = 0.37, *SE* = 0.14, *t* = 2.60, *p* = 0.009). The direct effect of individuals’ cellular inflammatory tendencies on present-focused decision-making was not significant (*β* = −0.03, *SE* = 0.09, *t* = −0.36, *p* = 0.72; Fig. 3); instead, the relationship between these two variables was mediated through current plasma levels of inflammation^47^. Overall, the model accounted for 12.9% of the variance in the latent present-focused decision-making variable.

**Table 1:** Descriptive statistics for observed variables.

Variable	*M* (*SD*)
BIS-11	2.12 (0.35)
DGI	6.61 (0.87)
Temporal Focus	64.17 (20.41)
Delay Discounting	6.60 (3.77)
Plasma IL-6	1.62 (1.43)
Plasma TNF-α	96 (0.31)
White Blood Cell Count	6.22 (1.85)
Stimulated IL-1β Release	1762.85 (1647.97)
Stimulated IL-6 Release	5549.86 (2871.59)
Stimulated TNF-α Release	1797.87 (1133.44)

*Note*. BIS-11 = Barratt Impulsiveness Scale; DGI = Delaying Gratification Inventory.

All cytokine measures were transformed prior to analyses; shown here are the raw values prior to transformation in pg/mL. White blood cell count was also transformed; shown here raw as number × 10^9^/L. Stimulated release values shown here collapsing across the four time points: 2, 24, 48, and 72 hours.

The pattern and significance of these relationships were not moderated by sex, and remained unchanged even when controlling for additional factors that might influence inflammation, present focus, or the relationship between the two (see Supplementary Information for results of these models)^48^. For these follow-up models, the effects of all tested covariates were controlled for in each path of the model. In addition, the pattern and significance of the results remained unchanged when removing white blood cell count from the model as an indicator of the latent current inflammation construct (see Supplementary Information for results of this model).

In addition to our primary statistical model, we ran two alternative models to test the directionality of the relationship between inflammation and present focus (see Supplementary Information for full results). This was important, given the cross-sectional nature of the current research and the possibility that harmful, present-focused behaviours drive inflammation rather than the reverse^49,50^. In each of these models, we examined whether behavioural correlates of present focus – such as smoking, alcohol intake, risky sexual behaviours, and adiposity – themselves predict inflammation (see Figure S1 and Figure S2 in Supplementary Information), thereby potentially providing alternative explanations for our results.

For both models, we first regressed smoking, alcohol consumption, sexual risk-taking, and BMI on the latent present-focused decision-making variable. For the first alternative model (see Figure S1 in Supplementary Information), we then regressed cellular inflammatory tendencies on each behavioural correlate of present focus, and regressed current inflammation on cellular inflammatory tendencies. Results revealed poor model fit (see Table S2 in Supplementary Information for fit statistics). The latent construct of present-focused decision-making predicted a higher likelihood of smoking, *β* = 0.27, *SE* = 0.12, *t* = 2.19, *p* = 0.03, and greater alcohol consumption, *β* = 0.42, *SE* = 0.11, *t* = 3.67, *p* < 0.001. There was a marginal positive relationship between present-focused decision-making and sexual risk-taking, *β* = 23, *SE* = 0.13, *t* = 1.87, *p* = 0.06. Present focus did not significantly predict BMI (*p* = 0.94), however. No behavioural correlate of present focus emerged as a significant predictor of cellular inflammatory tendencies (*p*s > 0.39). Cellular inflammatory tendencies, however, remained a significant predictor of current inflammation, *β* = 0.28, *SE* = 0.12, *t* = 2.40, *p* = 0.02.

For the second alternative model (see Figure S2 in Supplementary Information), we switched the order of cellular inflammatory tendencies and current inflammation in the model. Results again revealed poor model fit. None of the behavioural correlates of present focus predicted current inflammation, (*p*s > 0.18), and current inflammation was not a significant predictor of cellular inflammatory tendencies (*p* = 0.29). The results of these alternative models lend support to the proposed directionality of our hypothesized structural path model. Specifically, the behavioural correlates of present-focused decision-making did not significantly predict either levels of *in vivo* or *in vitro* inflammation.

At first blush, the lack of a relationship between present-focused behavioural outcomes (e.g., alcohol consumption, smoking, BMI) and inflammation may seem to contradict prior research, as well as our own theoretical model (see Fig. 1). However, it is important to note that great care was taken to try to minimize this link through our careful participant screening procedure (e.g., only those with a healthy BMI) and strict pre-session behavioural requirements. Indeed, all participants were required to abstain from inflammation-promoting behaviours (see Method for full list) for at least 48 hours prior to the testing session. These steps were taken to allow better isolation of the effects of inflammation on present-focused decision-making, while minimizing the strength of the reverse path. In addition to our strict pre-session screening criteria, that we recruited only healthy college-aged participants may also have contributed to the lack of relationship between present-focused behaviours and the inflammatory markers measured in the current research.

## Discussion

The results of the current research found key initial support for the hypothesis that inflammation – even at sub-clinical levels in healthy participants – may promote decision-making characterized by impulsivity, present focus, and an inability to delay gratification. These results suggest that the activities of the immune system may play an important role in shaping decision-making preferences, offering important new insights to research on temporal focus^1–6^, risk-sensitive foraging theory^25–28^, and the growing body of research demonstrating that the internal, physiological condition of the body plays an important role in modulating decision-making and behaviour^13,14,33^. Moreover, by potentially identifying a novel biological contributor to decision patterns that are known to promote myriad social and health problems^4–8^, the present results suggest exciting new possibilities for treatment and interventions aimed at improving personal and societal outcomes by targeting inflammation. For example, consistent with the present results, research conducted in non-human animals finds that neuroinflammation leads to increased self-administration of drugs like cocaine and opioids^40–42^. Accordingly, inflammatory pathways may be important targets for interventions seeking to improve outcomes for those with substance abuse disorders.

In addition to providing support for the hypothesized evolutionarily-grounded model, the results of the current research are consistent with recent theoretical work suggesting that inflammation plays a role in self-regulation, a construct closely related to present-focused decision-making^43^. This latter model posits that inflammatory activity may impair self-regulation, which is then hypothesized to alter developmental trajectories in ways that further reinforce inflammation and the inability to self-regulate. Although the current research provides initial support both for this model and our own, future research is needed to test additional predictions offered by these models. Experimental studies are also needed to make firm conclusions about the causal relationships between inflammation and present-focused decision-making proposed by these models.

The current findings also contribute to research examining the link between early life stress and proinflammatory tendencies^19^ on the one hand, and early life stress and present focus on the other^30,31^. The present results suggest that, rather than representing isolated, theoretically distinct correlates of early life adversity, these outcomes may be inextricably linked through the activities of the immune system. In addition to offering a mechanistic bridge between these seemingly disparate lines of inquiry, this suggests that being prone to inflammation (something that is observed among those who have experienced early life poverty^19^), may not itself fate an individual to subsequent present-focused decision-making. Instead, interventions targeted at reducing circulating levels of proinflammatory cytokines may sever the link between an individual’s propensity to inflammation and undesirable behaviours, improving outcomes for those from disadvantaged backgrounds.

The current research has important limitations. Although numerous steps were taken to help address the issue of directionality, the current research is cross-sectional in nature. Accordingly, it should be interpreted with care. This is of particular importance given the proposed bidirectional relationship between inflammation and present-focused decision-making. Indeed, others’ cross-sectional work has recently found that behavioural correlates of low self-control (e.g., adiposity and smoking) better predict inflammation than inflammation predicted self-control^50^. The differing pattern of results found between this previous study and the present study underscores the need for future research to examine relationships between inflammation and present-focused decision-making using experimental or longitudinal designs. Further, additional research is needed to examine the precise nature and neurobiological underpinnings of the link between inflammation and present focus, given that each are multifaceted constructs.

Nonetheless, the current research suggests that the activities of the immune system may play a key role in the development of decision-making strategies characterized by impulsivity, present focus, and an inability to delay gratification. These results may lay the groundwork for novel approaches to preventing and treating present-focused decision-making and its destructive impact on individuals and society.

## Methods

### Participants

Written informed consent was obtained from all participants, and the research was approved as compliant with ethical standards by the Texas Christian University Institutional Review Board (Approval #: 1411-117-1606). The current research was conducted in accordance with the relevant guidelines and regulations.

To help rule out the alternative explanation that harmful, present-focused behaviours drive inflammation – as some research suggests^50^ rather than the reverse path we predicted, we recruited only healthy, non-obese participants who were asked to abstain from behaviours that can contribute to inflammation (i.e., sex, exercise, smoking, and drug/alcohol use) for 48 hours prior to participating. Characteristics of the sample are shown in Table 2. Participants were 159 men and women recruited from Texas Christian University and the surrounding community (80 men, 79 women; *M*_age_ = 20.17 years, *SD* = 2.75). Sample size was determined by conducting an a priori power analysis using G*Power software (version 3.1.9^51^). Using the smallest effect size found for the relationship between inflammation and present-focused decision-making in our pilot data (*R*^2^ = 0.05) and a two-tailed *p*-value set at 0.05, we determined that we would need a total sample size of 152 participants in order to achieve 0.80 power. We collected 159 participants to prepare for the possibility of any data loss during the sample assay process.

**Table 2:** Characteristics of the sample (*N* = 159).

Sex: Men = 80; Women = 79
Age (17–30): *M* = 20.17, *SD* = 2.75
Race
White: 66.67% (*n* = 106)
Black: 4.40% (*n* = 7)
Hispanic: 15.09% (*n* = 24)
Asian: 6.29% (*n* = 10)
Multiracial/Other: 7.55% (*n* = 12)
Body mass index (17.8–29.8): *M* = 23.40 kg/m^2^, *SD* = 2.97
Exercise hours per week: *M* = 4.69, *SD* = 2.90
Typical hours of sleep: *M* = 6.83, *SD* = 1.32
Childhood SES (1–10): *M* = 5.97, *SD* = 2.07
Adult SES (1–10): *M* = 6.62, *SD* = 1.63

*Note*. All participants came in fasting and healthy. Participants were instructed not to consume alcohol, exercise, or take anti-inflammatory medications for two days prior to their testing session. All women in the sample were non-pregnant and not on hormonal contraceptives. Women’s sessions all took place 4–7 days after the start of their menstrual cycles.

Eligibility requirements included (1) being without history of chronic medical disorders, (2) being non-obese (body mass index [BMI] below 30), (3) not taking hormonal contraceptives (females), (4) being free from acute illness for at least two weeks prior to the date of participation, (5) being willing to abstain from steroidal and non-steroidal anti-inflammatory medications, exercise, and alcohol for at least two days prior to the session, and (6) being willing to fast the morning of the session. All women participated 4–7 days after the first day of their last menstrual period, to ensure that all were in the early follicular phase of their cycle. Participants were compensated with a choice of a $50 gift card or partial course credit.

### Procedure and materials

All testing sessions began at 7:30 AM, after each participant fasted for a minimum of eight hours. Upon receiving informed consent and ensuring that participants had followed the requirements of the study (e.g., abstaining from anti-inflammatory drugs, etc.), participants entered the lab in groups of 2–6, and sat at partitioned computer terminal stations. Participants completed all questionnaires and behavioural measures using Qualtrics online experimental survey software (Qualtrics, Provo, UT). After completing all survey measures, participants were ushered one at a time into an adjoining, private room where 85 mL of blood was drawn via venepuncture into heparinized (or EDTA-containing) Vacutainer® tubes (Becton-Dickinson, Franklin Lakes, NJ). After completion of the blood draw, participants were thanked, debriefed, and compensated.

### Measures of cellular inflammatory tendencies

The inflammatory tendencies of isolated peripheral blood mononuclear cells (PBMCs) were assessed by measuring *in vitro* release of IL-6, IL-1β, and TNF-α in response to both LPS stimulation and in the absence of stimulation. This measure captures a relatively stable index of the extent to which an individual’s white blood cells tend to release inflammatory factors in response to immunological challenge^19,52^.

PBMCs were isolated from whole blood using density gradient centrifugation in Ficoll® Paque Plus (Sigma-Aldrich, St. Louis, MO [GE Healthcare Life Sciences]), washed several times in Hank’s Balanced Salt Solution (HBSS; Caisson Labs, Logan, UT), counted, and plated into Falcon^TM^ 96-well tissue culture plates (Corning, Tewksbury, MA) in RPMI-1640 cell culture medium supplemented with 10% heat-inactivated foetal bovine serum, 2mM L-glutamine, 1 mM sodium pyruvate, 100 U of penicillin/mL, 100 µg of streptomycin/mL, and 0.25 µg of amphotericin B/mL (Caisson Labs, Logan, UT) at a density of 2.5 × 10^5^ cells/well, in a 200 µL volume. PBMCs were incubated for up to 3 days at 37 °C, 5% CO_2_, and 100% humidity. PBMCs were plated in media only and with 1 µg/mL of LPS, obtained from *Escherichia coli* (serotype 026:B6, Sigma-Aldrich, St. Louis, MO), in triplicate. Cell culture supernatants were harvested at 2, 24, 48, and 72 hours post-stimulation, then stored at −80 °C until assayed in duplicate for levels of IL-6, IL-1β, and TNF-α using a MILLIPLEX® MAP Human Cytokine Panel magnetic bead kit (EMD Millipore Corporation, Billerica, MA) with intra-assay coefficients of variation (CVs) of 8.20% (IL-6; Range: 2.81–19.41%), 6.97% (IL-1β; Range: 2.68–9.66%), and 5.98% (TNF-α; Range: 3.46–14.54%), and inter-assay CVs of 17.27% (IL-6; Range: 15.14–19.41%), 10.53% (IL-1β; Range: 10.45–10.62%), and 11.62% (TNF-α; Range: 11.56–11.68%). Supernatant cytokine levels were quantified using a Luminex MAGPIX® fluorescent detection system (Luminex, Austin, TX) and xPONENT® software (Version 4.2; build: 1324; Luminex, Austin, TX).

For each cytokine, spontaneous release (i.e., in the media-only condition) was subtracted from stimulated release at each time-point to compute an inflammatory tendency score. A score was computed for each cytokine (IL-6, IL-1β, and TNF-α) at each time-point (2, 24, 48, and 72 hours). Together, these items loaded onto a latent factor, comprising one’s cellular inflammatory tendencies.

It should be noted in regards to our proinflammatory cytokine measures that all cytokines – in this case IL-6 in particular – have pleiotropic effects and may not act in an exclusively proinflammatory fashion^53^. Nonetheless, because IL-1β, IL-6, and TNF-α have well-documented proinflammatory effects across many contexts^15–19,34^, we chose these cytokines as they together provide an index of inflammatory activity.

Due to a freezer failure on November 5, 2016, stimulated *in vitro* cytokine release samples were compromised for 32 participants. All compromised samples were excluded from assays and analysis. All other data from these participants were included in analyses, as these participants’ other biological samples were stored elsewhere. Missing data were handled per convention using the robust maximum likelihood estimation (MLR) in MPlus at the time of data analysis^45,46,54^. See Supplementary Information for more information.

### Measures of current inflammation

We first quantified current inflammation by examining plasma levels of the proinflammatory cytokines IL-1β, IL-6, and TNF-α. Plasma was collected in to EDTA-containing tubes, centrifuged, and immediately stored at −80° until assayed. Plasma was assayed using commercially available high-sensitivity enzyme-linked immunosorbent assay (ELISA) kits (R&D Systems, Minneapolis, MN) with intra-assay CVs of 3.58% (IL-6; Range: 2.72–5.57%) and 4.68% (TNF-α; Range: 3.09–6.79%), and inter-assay CVs of 13.91% (IL-6; Range: 9.82–17.80%) and 7.92% (TNF-α; Range: 2.80–13.53%). Fewer than 20% of participants’ plasma samples registered IL-1β levels above the lowest standard curve value (i.e., within detectable range). Thus, IL-1β values were excluded from all analyses (see Supplementary Information for additional details).

Samples were processed and quantified in duplicate, per manufacturer instruction, and plates were read at 490 nm with the correction wavelength set at 650 nm on a plate reader (BMG LabTech FLUOstar™ Omega, Cary, NC). Additionally, we measured total white blood cell count, which has been used in previous research as a marker of general systemic inflammation^49,55,56^. White blood cell count and type was assessed using electrical impedance in a haematology analyser (AC·T™ *5diff CP*, Beckman Coulter, Indianapolis, IN). Together, each of these items was loaded onto a latent construct of current inflammation.

### Measures of present-focused decision-making

Because present-focused decision-making is a multifactorial construct consisting of several different characteristics, such as acting without full reflection, present focus, and delay discounting^4,6,57^, four separate measures were collected in order to capture key components of the construct: two self-report scales and two behavioural assays.

First, participants responded to the Barratt Impulsiveness Scale (BIS-11), the gold standard measurement used in clinical and academic research studying the construct of impulsivity for over 50 years^57^. This scale consists of 30 statements describing cognitive and behavioural tendencies related to impulsivity. Participants rated how often they experienced each tendency, and a mean composite was computed, with a higher score indicating greater impulsivity (α = 0.85).

Participants also filled out the short-form Delaying Gratification Inventory^58^ to measure preference for immediate over delayed opportunities. The short-form DGI is comprised of 10 statements regarding the ability to delay gratification, for which participants rated how well each item described their tendencies. Relevant items were reverse-scored before being formed into a mean composite variable (α = 0.70), such that a higher score represented more difficulty delaying gratification (i.e., focus on immediate rewards).

Next, present focus was measured by presenting participants with a slider scale to indicate how “far away” certain periods of time felt^59^. The slider ranged from *very short* to *very long*, with 100 points of reference from the first endpoint to the last. Participants were asked, “How long from now does — feel?” for 1 day, 1 month, 3 months, and 6 months. A mean composite of perceived distance from each time period was computed (α = 0.85), with a longer perceived distance indicating a more present, as opposed to future, focus.

Finally, participants completed a monetary task in order to assess delay discounting, or the preference for smaller, immediate rewards over larger, delayed rewards^31^. Participants made 20 choices between two hypothetical rewards. Each of these dichotomous choices was presented in random order, with the following statement: “Do you want to get $__ tomorrow OR get $__ 33 days from now?” The “tomorrow” values were always smaller than the later values and the differences between the two values across the choices varied from $1 to $70. The total number of times a participant chose the immediate, smaller value was summed into a composite, with a higher score indicating a greater preference for smaller, immediate over larger, delayed rewards.

Scores from the BIS-11, DGI, and each of the behavioural assays (the temporal focus task and delay discounting task) loaded onto a single latent construct of present-focused decision-making. This construct served as our dependent measure. Refer to the Confirmatory Factor Analysis within the Supplementary Information for more details.

### Alternative explanations and tested covariates

In order to rule out alternative explanations for the relationship between inflammation and present-focused decision-making, several other variables shown to covary with present focus, inflammation, or both, were assessed. These included childhood and adult socioeconomic status, age, sex, body mass index (BMI), physical activity, sleep, stress, loneliness, and recent illness^48^. Childhood and adult socioeconomic status were measured using the MacArthur scale of subjective social status^60^. Physical activity was assessed by asking participants to answer the question, “How many hours of exercise do you do in a typical week?” Participants reported their typical sleep pattern by responding to the question, “How many hours of sleep do you get in a typical night?” Stress was assessed with the Perceived Stress Scale^61^, and loneliness was measured using the revised UCLA Loneliness Scale^62^. Finally, acute illness was measured by asking participants to rate agreement with the statement, “I am feeling sick today.” on a 7-point scale.

We also took measures of four present-focused behaviours or outcomes in order to test alternative models, thereby examining the proposed directionality of our hypothesized model. These included smoking, measured with the question, “Do you regularly smoke cigarettes?”, alcohol consumption, measured by asking participants to report how many alcoholic beverages they drink in a typical week, sexual risk-taking, measured with the behavioural subscale of the Revised Sociosexual Orientation Inventory^63^, and approximate adiposity measured by body mass index (BMI).

## Supplementary information

Supplementary Information for Inflammation Predicts Decision-Making Characterized by Impulsivity, Present Focus, and an Inability to Delay Gratification

## Data Availability

The datasets generated during and/or analysed during the current study are available in the Open Science Framework repository (DOI 10.17605/OSF.IO/SEBTZ).
