# Supplementary material for: Inflammation Predicts Decision-Making Characterized by Impulsivity, Present Focus, and an Inability to Delay Gratification
Source: Sci Rep. 2019 Mar 20;9:4928. doi: 10.1038/s41598-019-41437-1 (PMC6426921; doi:10.1038/s41598-019-41437-1)
Supplement: Supplementary file 1 — Supplementary Information for Inflammation Predicts Decision-Making Characterized by Impulsivity, Present Focus, and an Inability to Delay Gratification [file 41598_2019_41437_MOESM1_ESM.docx]

Supplementary Information for

Inflammation Predicts Decision-Making Characterized by Impulsivity, Present Focus, and an Inability to Delay Gratification

Jeffrey Gassen^1^*, Marjorie L. Prokosch^1^, Micah J. Eimerbrink^1^, Randi P. Proffitt Leyva^1^, Jordon D. White^1^, Julia L. Peterman^1^, Adam Burgess^1^, Dennis J. Cheek^2^, Andreas Kreutzer^3^, Sylis C. Nicolas^1^, Gary W. Boehm^1^, and Sarah E. Hill^1^

Correspondence to: j.gassen@tcu.edu

^1^Texas Christian University, Department of Psychology, 2955 S University Dr., Fort Worth, TX, United States of America

^2^Texas Christian University, Harris College of Nursing and Health Sciences, 2800 W Bowie St, Fort Worth, TX, United States of America

^3^Texas Christian University, Department of Kinesiology, 2800 W Bowie St, Fort Worth, TX, United States of America

**This file includes:**

Pilot data information

Extended results

Supplementary text

Supplementary Tables S1, S2, and S3

Supplementary Figures S1 and S2

**Pilot Data**

Hill and colleagues found initial support for these predictions in a correlational pilot study. The study examined the relationship between cellular proinflammatory tendencies (quantified by examining *in vitro* proinflammatory cytokine release in response to LPS), *in vivo* cytokine levels (quantified from participants’ plasma), and impulsivity (measured using BIS-11) in a sample of 67 students. The results of this pilot study found evidence of a positive relationship between cellular inflammatory tendencies, current inflammation, and impulsivity. Although these pilot data found initial support for our theoretical model, given the relatively small sample size and limited number of inflammatory and markers of present-focused decision-making, we sought to conceptually replicate these effects using a fully-powered, more robust study design.

**Extended Results**

**Confirmatory factor analysis**

We conducted a confirmatory factor analysis to test the fit of the proposed factor structure to the data. This initial step included loading indicators on the latent factors of current inflammation (plasma IL-6, plasma TNF-α, and white blood cell count), cellular inflammatory tendencies (stimulated release of IL-1β, IL-6, and TNF-α across time), and present-focused decision-making (BIS-11, DGI, temporal focus, and delay discounting). All factor loadings were significant, revealing significant relationships between each observed variable and the overall construct. All requirements for model fit were met, per convention (Kline, 2016). Factor loadings are displayed in Table S1 and model fit statistics are displayed in Table S2. Because the factor loadings for the self-report measures were stronger than the behavioral measures, we also tested a model with two latent factors of present-focused decision-making: self-report (BIS-11 and DGI) and behavioral (temporal focus and delay discounting). We found that the two-factor model did not improve model fit, *χ*^2^(3) = 3.85, *p* > .05, and the two present-focused decision-making factors were strongly correlated (*r* = .50, *p* = .008). Given these results, we proceeded using the hypothesized single factor model.

**Covariates model**

To test whether the relationship between inflammation and present-focused decision-making was robust to controlling for covariates, we regressed each latent factor on age, report of recent illness, sleep, exercise, BMI, childhood and adult socioeconomic status, loneliness, and stress, in addition to the paths specified in the hypothesized model. See Table S2 for model fit statistics. As with the model excluding covariates, results revealed that more pronounced proinflammatory cellular tendencies predicted higher levels of current inflammation, *β* = .22, *SE* = .10, *t* = 2.16, *p* = .03, and higher current inflammation predicted greater present-focused decision-making, *β* = .33, *SE* = .15, *t* = 2.14, *p* = .03. The direct effect of cellular inflammatory tendencies on present-focused decision-making remained non-significant, *p* = .48. A separate model was estimated to test for sex differences in the relationship between inflammation and present-focused decision-making, as well as to assess the impact of controlling for sex on this relationship. Results revealed no significant interactions with sex for any path (*p*s > .44). Further, the relationship between cellular inflammatory tendencies and current inflammation remained significant, (*p* = .04), as did the relationship between current inflammation and present-focused decision-making, (*p* = .002), while controlling for sex.

**Post-hoc power analysis**

To assess the power achieved with our current sample size in detecting the effects estimated in the primary model, we conducted a Monte Carlo simulation in MPlus (e.g., see Muthén & Muthén, 2002 for summary) with 10,000 repetitions. We specified the number of observations as our actual sample size, and fixed the expected parameter estimates as the standardized coefficients found in the primary model.

This simulation revealed negligible parameter and standard error biases (all < 5%) as well as high percentage of replications for which the the 95% confidence interval contained the true parameter value (> 93% for all parameters in model). Results revealed sufficient power to detect the significant effects for each parameter (i.e., at least .80 power) with the exception of the observed variables of delay discounting (.78 power) and temporal focus (.74 power) as indicators of the latent factor of present-focused decision-making.

We repeated the simulation after decreasing our specified *N* to coincide with the missing cellular inflammatory tendencies data. After this modification, we no longer had adequate power to detect the relationship between cellular inflammatory tendencies and current inflammation (.66 power). No other parameters in the model were affected.

**Hypothesized model without white blood cell count**

Because white blood cell count is a less direct measure of inflammation than levels of proinflammatory cytokines, we analyzed the hypothesized path model a second time after removing white blood cell count as an observed variable. Thus, the latent factor of current inflammation was comprimsed only of levels of IL-6 and levels of TNF-α. Results revealed that removing white blood cell count from the analysis did not change the pattern or significance of the results. Specifically, greater proinflammatory cellular tendencies predicted higher levels of current inflammation, , *β* = .27, *SE* = .13, *t* = 2.04, *p* = .04, and higher levels of current inflammation predicted greater present-focused decision-making, *β* = .32, *SE* = .11, *t* = 2.83, *p* = .005.

**Supplementary Text**

**Measures of cellular inflammatory tendencies**

We conducted an exploratory multivariate analysis of variance (MANOVA) in order to determine whether our key observed variables differed in any systematic way between participants for whom we had full stimulated cytokine data and those for whom we lost samples as a result of a freezer failure (*N* = 32). Descriptive statistics are shown in Table S3. Results revealed no significant differences between the groups on any of the key observed variables (*F*s < 3.32, *p*s > .07). Because the delay discounting measure approached significance in our MANOVA (*p* = .07), we conducted a follow-up univariate analysis of variance (ANOVA) which again revealed no significant difference, *F*(1, 157) = 2.39, *p* = .12. Thus, we concluded that there were no significant systematic differences between those affected and unaffected by the freezer failure.

**Measures of current inflammation**

We assayed plasma IL-1β using high-sensitivity ELISAs purchased from R&D Systems (Minneapolis, MN, catalog #: HSLB00D). In the past, we had successfully quantified IL-1β in healthy human plasma using a since-discontinued version of the assay (catalog #: HSLB00C). However, likely due to a change in the capture antibody, fewer than 20% of plasma samples in the current study registered IL-1β levels above the lowest standard curve value (i.e., within detectable range). These data were therefore not used in any analyses.

**References**

1. Muthén, L. K., & Muthén, B. O. How to use a Monte Carlo study to decide on sample size and determine power. *Struct. Equ. Model.* **9**, 599–620 (2002).

| **Table S1. Results of confirmatory factor analysis.** | | |  |  | |  |
| --- | --- | --- | --- | --- | --- | --- |
|  | Estimate | | *SE* | *p* | | *R*^2^ |
| Present-focused Decision-making |  | |  |  |  | |
| DGI | | .79 | .10 | <.001 | .63 | |
| BIS-11 | | .68 | .10 | <.001 | .47 | |
| Temporal Focus | | .22 | .09 | .02 | .05 | |
| Delay Discounting | | .24 | .09 | .007 | .06 | |
| Current Inflammation | |  |  |  |  | |
| Plasma IL-6 | | .87 | .14 | <.001 | .75 | |
| Plasma TNF-α | | .37 | .09 | <.001 | .14 | |
| White Blood Cell Count | | .41 | .13 | .001 | .17 | |
| Cellular Inflammatory Tendencies | |  |  |  |  | |
| Stimulated IL-1β Release | | .88 | .04 | <.001 | .78 | |
| Stimulated IL-6 Release | | .74 | .05 | <.001 | .55 | |
| Stimulated TNF-α Release | | .88 | .03 | <.001 | .77 | |
|  | |  |  |  |  | |

*Note.* Standardized estimates listed for final CFA model. Standard errors, *p*-values, and *R*^2^ shown. *R*^2^  refers to the variance in each observed variable explained by the latent factor. BIS-11 = Barratt Impulsiveness Scale; DGI = Delaying Gratification Inventory.

| **Table S2. Summary of model fit indices.** | | | | |
| --- | --- | --- | --- | --- |
|  |  |  |  |  |
| Model | *χ*^2^(*df*) | CFI | RMSEA | SRMR |
| CFA Model | 40.64(32) | .97 | .02 [.00, .04] | .05 |
| Hypothesized Model | 40.64(32) | .97 | .02 [.00, .04] | .05 |
| Covariates Model | 202.16(95)* | .74 | .05 [.04, .05] | .06 |
| Alternative Model 1 | 147.37(72)* | .79 | .04 [.03, .05] | .08 |
| Alternative Model 2 | 154.97(72)* | .77 | .04 [.03, .05] | .08 |
|  |  |  |  |  |

*Note.* CFA = confirmatory factor analysis; CFI = comparative fit index;
RMSEA = root mean square error of approximation; SRMR = standardized root mean square residual. Fit statistics not available for sex differences model. **p* < .05

**Table S3. Descriptive statistics for lost stimulated cytokine sample analyses.**

Included (*N* = 127) Lost (*N* = 32)

| Variable | *M* (*SD*) | *M* (*SD*) |
| --- | --- | --- |
|  |  |  |
| BIS-11 | 2.13 (.37) | 2.10 (.26) |
| DGI | 6.60 (.87) | 6.64 (.85) |
| Temporal Focus | 65.37 (19.95) | 61.38 (22.53) |
| Delay Discounting | 6.83 (3.49) | 5.82 (4.57) |
| Plasma IL-6 | 1.71 (1.52) | 1.35 (1.00) |
| Plasma TNF-α | .98 (.33) | .90 (.22) |
| White Blood Cell Count | 6.27(1.99) | 6.04 (1.22) |
|  |  |  |

*Note.* BIS-11 = Barratt Impulsiveness Scale; DGI = Delaying Gratification Inventory.

All cytokine measures were square-root transformed prior to analyses; shown here are the raw values prior to transformation in pg/mL. White blood cell count was also transformed; shown here raw as number × 10^9^/L.


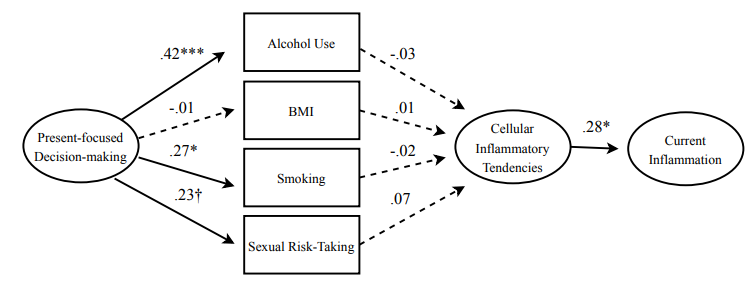


**Figure S1***.***Alternative model testing relationships between present-focused behaviors and cellular inflammatory tendencies.** Final model shown with standardized estimates. Dotted lines denote *p* > .08. Circles denote latent constructs confirmed in the CFA. ****p* < .001; ***p* < .01; **p* < .05; †*p <* .08.


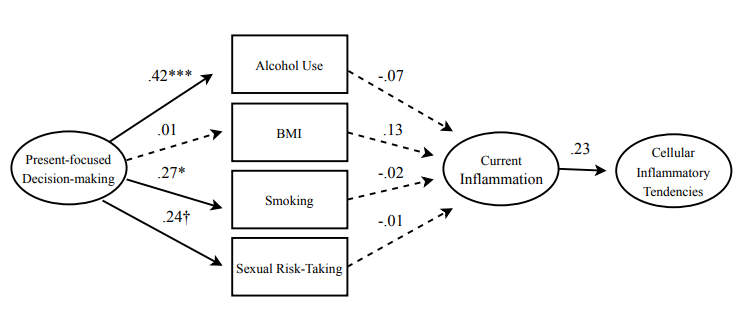


**Figure S2. Alternative model testing relationships between present-focused behaviros and current inflammation.** A Final model shown with standardized estimates. Dotted lines denote *p* > .08. Circles denote latent constructs confirmed in the CFA. ****p* < .001; ***p* < .01; **p* < .05; †*p <* .08.
